# Incidence and risk factors for retinal detachment after cataract surgery: a comparison between trainee and experienced surgeons

**DOI:** 10.1186/s40942-025-00793-z

**Published:** 2026-01-08

**Authors:** Thiago Meister, Helena Gschwendtner, Eric Koji Azuma Watanabe, Amanda Granero de Castro, Ana Beatriz Ferreira do Amaral Antunes, Luís Expedito Sabage, Carlos Augusto Moreira-Neto

**Affiliations:** 1 Hospital de Olhos do Paraná, Alameda Pres. Taunay, 483 - Batel, Curitiba, 80420-180 PR Brazil; 2 Faculdade de Medicina de Bauru, Universidade de São Paulo, Al. Dr. Otávio Pinheiro Brisolla 9-75, Vila Universitária, Bauru, 17012-901 SP Brazil

**Keywords:** Cataract surgery, Rhegmatogenous retinal detachment, High myopia, Lattice degeneration, Surgical experience, Learning curve, Complications risk

## Abstract

**Background:**

To evaluate the incidence and independent risk factors for rhegmatogenous retinal detachment (RRD) following cataract surgery within a single institutional cohort, with a specific focus on surgeon experience (training vs. experienced surgeons), and to contextualize findings with large-scale population-based studies.

**Methods:**

Retrospective cohort study based on data from electronic medical records from 23,642 eyes that underwent phacoemulsification between 2018 and 2021 at a tertiary ophthalmology center, 13,294 of which were operated by training surgeons (TS) and 10,348 by experienced surgeons (ES). Postoperative outcomes were assessed within a fixed follow-up period of up to 12 months. Variables included surgeon experience, age, sex, high myopia (HM), and lattice degeneration (LD). Eyes with HM + LD were analyzed descriptively but excluded from multivariable modeling due to collinearity. Exclusion criteria: patients aged < 40 years, previous RRD, tractional or exudative retinal detachment, diabetic retinopathy, and intraoperative posterior capsule rupture. Firth’s penalized logistic regression was used to identify independent risk factors for RRD.

**Results:**

Among all included eyes, 16 (0.07%) developed RRD within one year after cataract surgery, including 14 (0.11%) among TS and 2 (0.02%) among ES. A significantly higher adjusted risk for TS (OR: 5.67; 95% CI: 2.49–12.92; *p* < 0.001). Adjusted analysis confirmed HM (OR 12.04; 95% CI 4.25–34.09; *p* < 0.001) and LD (OR 12.78; 95% CI: 3.58–45.65) as strong independent risk factors. Male sex showed a modest but statistically significant association with RRD (adjusted OR 1.92; 95% CI 1.03–3.57; *p* = 0.039). Age showed a heterogeneous association with RRD: compared with patients < 60 years, those aged ≥ 70 years had significantly lower adjusted odds, whereas no significant difference was observed for the 61–70-year group.

**Conclusions:**

Surgeon experience is an independent and clinically relevant determinant of RRD risk after cataract surgery, even in uneventful procedures. This study represents the first single-center analysis including more than 20,000 eyes to isolate the impact of surgical experience within a standardized institutional setting while controlling for key ocular and demographic factors. These findings highlight the importance of structured supervision and training strategies to mitigate postoperative retinal complications, particularly in high-risk eyes.

**Supplementary Information:**

The online version contains supplementary material available at 10.1186/s40942-025-00793-z.

## Introduction

Several risk factors have been consistently associated with the development of RRD after phacoemulsification, including HM, LD, posterior capsular rupture, and prolonged surgical time. Additionally, patient-related factors such as younger age and male sex have been linked to increased susceptibility, as demonstrated in large-scale studies such as the IRIS Registry and population-based cohorts from France and Sweden. These studies reported a one-year incidence of RRD ranging from 0.21% to 0.25%, identifying HM and LD as key risk factors while underscoring demographic and biometric influences [1–4].

Despite the robustness of these large datasets, they often lack granular analysis regarding intraoperative variables, particularly the direct impact of surgeon experience on RRD risk. Although surgeon experience is generally recognized as a critical determinant of surgical outcomes, its isolated impact on the development of RRD following cataract surgery remains underexplored. Current literature presents conflicting findings, with many studies frequently reliant upon small sample sizes or multicenter data with inherent variability. This heterogeneity hinders the interpretability and generalizability of results [1–3, 5].

In this context, the present study aims to evaluate the incidence and risk factors for RRD following uneventful cataract surgeries performed by residents and fellows in training (training surgeons – TS) compared with those conducted by experienced surgeons (ES) within a single-center Brazilian ophthalmology center. Additionally, it seeks to contextualize these findings within the landscape of large-scale international studies to clarify the role of surgical experience as an independent factor in RRD risk, providing evidence to inform surgical training policies and patient safety strategies.

To our knowledge, this is the first single-center study with a sample size exceeding 20,000 eyes to directly compare training and experienced surgeons in terms of RRD incidence following uneventful cataract surgeries while adjusting for relevant ocular and demographic confounders such as HM, LD, sex, and age through multivariable logistic regression.

## Methods

This was a retrospective cohort study based on the analysis of electronic medical records of patients who underwent cataract surgery (phacoemulsification) between January 1, 2018, and December 31, 2021, at a Brazilian ophthalmology center. Surgeries were performed in two settings: the public health unit (SUS), where TS operated under supervision, and the private unit, where ES carried out procedures independently. ES were defined as ophthalmologists who had previously performed more than 1,500 independent cataract surgeries and had several years of post training clinical practice, ensuring a high level of surgical proficiency and consistency. TS consisted of ophthalmology residents in the surgical training phase, performing cataract surgery under direct supervision, with an average cumulative experience of approximately 100 cataract surgeries per trainee at the time of the procedures. The results were contextualized with global data from the IRIS Registry Study [1].

All cataract surgeries were performed using standardized surgical techniques; however, different phacoemulsification platforms were employed according to the clinical setting. Surgeries performed by TS were conducted using the Laureate^®^ phacoemulsification system (Alcon Laboratories, Fort Worth, TX, USA), whereas ES utilized the Centurion^®^ Vision System (Alcon Laboratories, Fort Worth, TX, USA).

Clinical and surgical information was extracted via Gesthor^®^ electronic medical records software, and all the data were deidentified prior to analysis. The study adhered to the tenets of the Declaration of Helsinki, and informed consent was waived due to its retrospective nature. Cataract surgeries performed by TS and ES were identified from surgical schedules, and demographic information (age and sex) was retrieved by corresponding patient identifiers. Clinical variables, including postoperative RRD, HM, and LD, were reviewed and categorized according to electronic medical records.

The variables collected included patient age, sex, the presence of HM, LD, and the combination of both (HM + LD), as well as the surgeon’s level of experience. HM was defined as greater than 6 diopters on the basis of preoperative refraction records. Axial length measurements were not uniformly available in a structured format across the electronic medical records. In this retrospective dataset, the identification of high myopia relied on preoperative clinical documentation, where high myopia was consistently recorded via refractive-based terminology.

LD was recorded as a binary variable (present or absent), as the extent of lattice involvement was not classified in the available medical records. The primary outcome was the occurrence of RRD within one year post-operatively. All extracted data were organized in a structured spreadsheet for statistical analysis.

Inclusion criteria encompassed all eyes undergoing cataract surgery in two distinct units: the SUS and the private unit. Exclusion criteria were patients under 40 years of age; a history of previous RRD, tractional retinal detachment (TRD) or exudative retinal detachment (ERD); diabetic retinopathy; and posterior capsule rupture (PCR) as an intraoperative complication. No patients requiring additional intraoperative procedures, such as suturing due to lens subluxation or management of acute glaucoma attacks, were identified in the study cohort. For the purpose of this study, an “uneventful procedure” was defined as cataract surgery that did not involve any intraoperative events listed in the exclusion criteria, ensuring a homogeneous cohort for comparison of surgeon groups under standard surgical conditions, without the influence of major intraoperative complications.

Statistical analysis was conducted using IBM SPSS Statistics for Windows, version 30.0 (IBM Corp., Chicago, IL, USA). Descriptive statistics were used to characterize the sample, and univariate analyses were initially performed to identify associations between RRD and the collected variables. Subsequently, given the low number of RRD events, multivariable analysis was performed via Firth’s penalized logistic regression, which is appropriate for rare-event outcomes and reduces small-sample bias and separation [6, 7]. Age was modeled via clinically relevant categorical contrasts (< 60 years as reference), rather than multiple stratified age categories, to avoid unstable estimates and create a reference for comparison. The multivariable models included surgeon experience, high myopia, lattice degeneration, sex, and age. The results were expressed as adjusted odds ratios (OR) with 95% confidence intervals (CI).

## Results

A total of 23,642 eyes were included in the study, with 13,294 (56%) operated by TS at the SUS unit and 10,348 (44%) operated by ES at the private unit. Rigorous measures, including the use of encryption and deletion of information after collection, were adopted to ensure data security and confidentiality.

Among all the eyes included, 16 cases of RRD were identified, corresponding to an incidence of 0.07%. Among the 13,294 eyes operated by TS, 14 cases of RRD were identified, corresponding to an incidence of 0.11%. In contrast, surgeries performed by ES resulted in 2 cases of RRD among the 10,348 eyes, yielding an incidence of 0.02% (*p* < 0.001; crude OR 5.45).

Among the total number of eyes, 109 (0.46%) were identified as having HMs: 68 patients (62%) operated by TS, of whom 5 (7.4%) developed RRD after phacoemulsification, and 41 patients (38%) operated by ES, where no RRD cases were observed. Statistical analysis confirmed a highly significant association (*p* < 0.001; crude OR 95).

For LD, 34 patients (0.15%) were identified. Among these patients, 14 patients (41%) operated by TS, of whom 1 (7.1%) developed RRD after phacoemulsification, and 20 patients (59%) operated by ES, with no RRD cases recorded in this group (*p* < 0.001; crude OR 78.5).

Additionally, 16 (0.07%) patients presented with an association of LD and HM, of whom 9 (0.04%) were operated by TS and 7 were operated by ES. Three (33.3%) of these patients developed RRD following surgery performed by TS, whereas no RRD cases were recorded in the ES group. (*p* < 0.001; crude OR 603). Crude comparisons are summarized in Table 1.

**Table 1:** Incidence of RRD within 1 year after cataract surgery by risk factor and comparison between TS and ES

Group		Cataract Procedures	RRD after Cataract Surgery			
		*N*	*N*	Incidence rate (%)	*P* Value (Chi-square Test)	Odds Ratio IC 95%
All procedures		23,642	16	0.07	-	-
	TS	13,294	14	0.11	< 0,001	5.45
	ES	10,348	2	0.02		
High Myopia	TS	68	5	7.4	< 0,001	95
	ES	41	0	0		
Lattice Degeneration	TS	14	1	7.1	< 0,001	78.5
	ES	20	0	0		
High Myopia + Lattice Degeneration	TS	9	3	33.3	< 0,001	603
	ES	7	0	0		

To determine whether these associations persisted after adjustment for potential confounders and to identify independent risk factors for RRD following phacoemulsification, a multivariable analysis using Firth’s penalized logistic regression was performed. This approach was chosen due to the low number of RRD events and the rare-event nature of the outcome.

The multivariable model included surgeon experience (TS vs. ES), high myopia, lattice degeneration, sex, and age; and was modeled using clinically relevant categorical contrasts (< 60 years as the reference category). The combined variable HM + LD was excluded from the multivariable analysis due to collinearity and sparse data.

After adjustment, surgeries performed by TS were independently associated with a significantly increased risk of RRD, with an adjusted OR of 5.67 (95% CI 2.49–12.92; *p* < 0.001), confirming the robustness of the association between surgeon experience and postoperative RRD.

High myopia was also independently associated with an increased risk of RRD (adjusted OR 12.04; 95% CI 4.25–34.09; *p* < 0.001), as was lattice degeneration (adjusted OR 12.78; 95% CI 3.58–45.65; *p* < 0.001).

Male sex demonstrated a modest association with RRD, with an adjusted OR of 1.92 (95% CI 1.03–3.57; *p* = 0.039).

Regarding age, patients aged 61–70 years did not demonstrate a statistically significant difference in RRD risk compared with those younger than 60 years (adjusted OR 0.63; 95% CI 0.33–1.19; *p* = 0.155). In contrast, patients aged 70 years or older had a significantly lower relative risk of RRD compared with the reference group (adjusted OR 0.11; 95% CI 0.05–0.29; *p* < 0.001).

For descriptive purposes, we examined the age distribution of RRD cases across the two surgical settings. Patients aged over 70 years represented the majority of cases (52%), with 30% originating from the SUS unit and 22% from the private unit. The 61–70 age group accounted for 34% of the cases (20% SUS, 15% Private), followed by the 51–60 age group, with 10% of the cases (even split between SUS and Private). The 40–50 age group contributed 2% of the cases (1% per unit), and only a single RRD case (1%) was observed in a patient under 40 years of age, occurring in the private unit cohort.

A detailed summary of the multivariable Firth penalized logistic regression analysis, including adjusted odds ratios, confidence intervals, and significance levels, is presented in Table 2.

**Table 2:** Multivariable Firth penalized logistic regression analysis for RRD within 1 year after cataract surgery

Predictor	95% Confidence Interval			
	Odds ratio	Lower	Upper	*p*
Training surgeons x Experienced Surgeons:				
Training Surgeons	5.67	2.49	12.92	< 0.001
High Myopia	12.04	4.25	34.09	< 0.001
Lattice Degeneration	12.78	3.58	45.65	< 0.001
Age 61–70 vs. <60	0.63	0.33	1.19	0.155
Age ≥ 70 vs. <60	0.11	0.05	0.029	< 0.001
Sex:				
Male	1.92	1.03	3.57	0.039

## Discussion

This study presents a unique methodological contribution. Unlike previous multicenter registries and comparative cohorts, this is the first report conducted within a single institution with a large surgical volume to apply multivariable logistic regression to assess surgeon experience (TS vs. ES) while controlling for known ocular risk factors (HM, LD) and demographic variables (sex, age), allowing for internal consistency in protocols and follow-up and offering a refined and institutionally controlled perspective on RRD risk after cataract surgery.

Our study revealed an incidence of 0.11% among TS procedures and 0.02% among ES procedures, which aligns with the observed trend that surgical experience significantly influences outcomes.

A comparative analysis with major studies contextualizes our findings within the international literature (see Additional File 1). The IRIS Registry and Daien et al. provide large-scale population-based incidence estimates for RRD following cataract surgery (0.21% and 0.25%, respectively), identifying HM and LD as significant risk factors but without accounting for surgeon experience [1, 2, 5]. Thylefors et al. similarly reported RRD incidence rates stratified by age in a Swedish cohort, reinforcing age as a relevant factor [4]. In contrast, Khatibi et al. specifically addressed surgeon experience, reporting a 0.76% RRD incidence in TS-performed surgeries but lacked multivariate adjustment for ocular risk factors, had a smaller sample size (3,871 eyes), combined different surgical techniques across different institutions, and reported findings contrasted with historical data from different studies on ES, limiting the interpretability of their findings [3].

Our study, despite the smaller cohort compared with large registries, demonstrated a lower overall RRD incidence (0.07%) but uniquely showed a significant, independently adjusted 5.67-fold higher risk of RRD in surgeries performed by TS, estimated using Firth’s penalized logistic regression, even after controlling for HM, LD, and sex. This methodological rigor enhances the reliability of our findings, positioning our study, to our knowledge, as the first in the literature to critically isolate and quantify the impact of surgeon experience on RRD risk within a controlled single-center environment, which enhances the reliability and internal validity of our data and provides evidence that is both clinically relevant and directly applicable to surgical training programs.

HM demonstrated a strong independent association with RRD, with an adjusted OR of 12.04 (95% CI: 4.25–34.09; *p* < 0.001). This aligns with IRIS Registry findings (OR 1.2 for HM) and a Taiwanese cohort that analyzed 9,388 eyes undergoing cataract extraction and reported an 8-year cumulative risk of 6.14% in eyes with axial length ≥ 26 mm [1, 5, 8]. Although the magnitude of the association observed in our study is greater than that reported in large registries, this likely reflects the very low prevalence of high myopia in our cohort (0.46%), which is a pattern that is consistent with population-based data in older surgical populations rather than a divergence in risk direction [9].

Similarly, previous epidemiologic studies have demonstrated that high myopia is relatively uncommon in individuals aged 60 years and older [9]. Notably, high myopia was classified on the basis of refractive error rather than axial length, which may introduce some degree of misclassification and should be considered when interpreting the magnitude of the observed association.

Similarly, LD also emerged as a strong independent risk factor (adjusted OR 12.78; 95% CI: 3.58–45.65; *p* < 0.001). This result corroborates findings from the IRIS data (OR 10.53; 95% CI: 9.82–11.28; *p* < 0.001) and reinforces the role of LD as a major risk factor for postoperative RRD [1]. According to the univariate analysis, the combination of HM and LD was associated with the highest crude risk in our analysis, emphasizing the synergistic risk when these factors coexist, especially in eyes managed within training environments. However, owing to collinearity and sparse data, this combined variable was not included in the multivariable model.

Differences in effect magnitude compared with multicenter registries are expected. IRIS estimates reflect aggregated risk across millions of surgeries performed in diverse clinical settings, with heterogeneity in patient profiles, surgical techniques, and postoperative surveillance [1]. In contrast, our study provides a more focused analysis of two specific surgical environments within a Brazilian ophthalmology center: a training center and a private center with ES. The higher odds ratios observed in this study, which were derived from a single training center, represent localized, institution-level associations rather than universal risk estimates and likely reflect contextual factors such as learning-curve effects and patient selection biases.

In this context, smaller samples may amplify the apparent impact of high myopia and lattice degeneration within a learning environment, where surgical risk is inherently greater. In such settings, sparse data bias may arise, a well-recognized phenomenon in epidemiological research whereby rare events and low-prevalence exposures can yield inflated or unstable odds ratio estimates. To mitigate this limitation, we applied Firth’s penalized logistic regression, an approach specifically designed to address sparse data bias and stabilize effect estimates in small-sample and rare-outcome settings. In contrast, large registries such as the IRIS Registry dilute these effects through scale, resulting in more stable population-level risk estimates [1, 10].

A multitude of factors may be associated with the mechanisms of RRD in TS cases. These include unrecognized zonular stress, surgical time, eye manipulation, wound construction and energy levels during surgery. It is important to acknowledge the potential influence of socio-economic factors on the observed higher rate of RRD. Patients utilizing the public healthcare system may tend to seek treatment at a later stage compared to those in private healthcare settings [11, 12]. Low-income populations are more vulnerable to previously undetected trauma and may exhibit distinct postoperative care patterns due to limited information and the physical demands of manual labor [13, 14].

Additionally, follow-up variability represents an important limitation. Even with a single-center design, differences in adherence between publicly treated patients (TS group) and privately treated patients (ES group) may influence the likelihood of detecting retinal tears or early RRD. Barriers to follow-up in the public system could allow undiagnosed retinal tears to progress outside the one-year observation period, partially explaining our lower overall RRD incidence (0.07%) than that reported in large registries [2, 15, 16]. Furthermore, some RRDs may have presented outside our institution or following retinal examinations performed by nonretinal specialists, resulting in potential under recognition. Although inherent to retrospective designs, this limitation likely had minimal impact given our uniform postoperative pathway.

Contrasting TS and ES within the same institutional environment, we found a 5.67-fold increased risk of RRD in TS procedures (adjusted OR 5.67; 95% CI: 2.49–12.92; *p* < 0.001) even after adjustment for patient-level factors, reinforcing surgical experience as an independent protective factor against postoperative RRD. These findings highlight that, even under comparable institutional conditions, surgical experience exerts a measurable influence on postoperative retinal outcomes.

The role of posterior capsule rupture (PCR) is particularly relevant in this context [17]. PCR is one of the strongest known intraoperative predictors of pseudophakic RRD, often overshadowing more subtle experience-related effects [18]. For this reason, we intentionally restricted our analysis to uneventful procedures to isolate the intrinsic impact of surgeon experience. Evidence from Day et al. supports this rationale: in the UK National Ophthalmology Database, pseudophakic retinal detachment occurred earlier both after cataract surgeries performed by trainees and after surgeries complicated by PCR, reported as independent associations [10].

Although the study did not evaluate an interaction between these two factors, their findings reinforce that postoperative retinal behavior is shaped by multiple layers of surgical complexity—including but not limited to, surgeon experience. By restricting the present analysis to surgeries free of PCR, we sought to minimize confounding factors and allow a clearer interpretation of the experience-related risk within our institution [10].

Several limitations must be considered when interpreting our findings. First, surgical time was not available in our dataset. A longer operative duration is known to increase intraoperative manipulation and vitreoretinal traction, particularly in the early stages of the learning curve, and has been associated with higher postoperative complication rates, including RRD. Incorporating surgical time in future analyses would help clarify the extent to which operative duration mediates the association between training status and RRD [19–22].

Additionally, posterior vitreous detachment (PVD) status could not be assessed. Although PVD is a recognized risk factor for retinal breaks and RRD after cataract surgery due to increased vitreous syneresis, its documentation is notoriously inconsistent in retrospective datasets, including large registries such as IRIS [1, 23, 24]. Reliable identification of PVD typically requires standardized protocols rarely present in routine cataract workflows. Prospective studies with systematic PVD assessment are needed to better delineate its independent contribution [1, 23, 25, 26].

In addition, differences in phacoemulsification platforms between the two surgical settings may have contributed to the variability in surgical efficiency and intraocular stability. Trainee surgeons operate using the Laureate^®^ system (Alcon), whereas experienced surgeons primarily use the Centurion^®^ platform (Alcon), which incorporates active fluidics technology and may offer improved chamber stability and energy modulation. Although both systems are widely used and considered safe, such differences could partially influence operative dynamics, particularly during the learning curve [27, 28].

Surgeon expertise also varies substantially across studies. Multicenter registries such as IRIS include thousands of surgeons with heterogeneous skill levels, which can dilute the ability to isolate the effects of surgical experience. In contrast, our internal comparison revealed a 5.67-fold increased RRD risk in TS cases within the same institutional environment, underscoring experience as an independent and clinically meaningful determinant of postoperative RRD [1–3].

The role of prophylactic laser treatment in these patients also warrants further attention, as prophylactic intervention could significantly affect RRD incidence in high-risk patients. Additionally, heterogeneity in protocols regarding prophylactic laser treatment for LD can influence reported RRD incidences across studies. While some centers advocate routine prophylactic photocoagulation in eyes with LD prior to cataract surgery, others may adopt less conservative approaches, leading to variable risk exposure in patient cohorts [16]. The IRIS and Daien studies do not specify prophylactic laser utilization rates, limiting comparisons [1, 2].

The intervention of ES in complex cases performed by TS may have also influenced outcomes, as ES may assume control of procedures initiated by TS when complications/difficulties arise. While essential for patient safety, this dynamic may underestimate complication rates attributable specifically to TS and attenuate measurable differences between groups. The variability in TS autonomy and supervision intensity across institutions further complicates comparisons with multicenter studies [20–22, 29]. Large-scale studies such as IRIS do not account for these nuances, whereas Khatibi et al. compared TS and ES outcomes without multivariate adjustment [1, 3].

In our penalized multivariable model, age showed a nonuniform association with RRD. Compared with patients < 60 years (reference for statistical model), patients aged 61–70 years were not significantly associated with RRD (adjusted OR 0.63; 95% CI 0.33–1.19; *p* = 0.155), whereas patients aged ≥ 70 years had significantly lower adjusted odds (adjusted OR 0.11; 95% CI 0.05–0.29; *p* < 0.001). These findings differ from those of the IRIS Registry, which reported substantially higher RRD risk among younger patients [1]. This discrepancy likely reflects differences in cohort structure and case mix between large multicenter registries and our single-center setting, as well as the limited number of RRD events available for age-stratified inference (*n* = 16).

Although Firth’s penalized logistic regression was applied to mitigate sparse data bias and stabilize estimates in this rare-event context [7], age-specific associations in small-event datasets should still be interpreted cautiously. The age distribution in our population also differs markedly from that of large registries: most RRD cases in our study occurred in older adults—reflecting local patterns of cataract surgical access in the public system—whereas younger patients are more frequently represented in private settings and multicenter databases. These differences in demographic structure and study design likely contribute to the divergent age-related findings [1, 2, 5, 8].

Male sex was independently associated with RRD in our multivariable analysis (adjusted OR 1.92; 95% CI 1.03–3.57; *p* = 0.039), which is consistent with population-based studies reporting a greater susceptibility to RRD among men [2, 4, 5, 8].

Overall, these limitations illustrate the challenges of precisely characterizing demographic associations with RRD in small institutional cohorts. Our findings indicate that, even after excluding major intraoperative complications, surgical experience independently influences RRD risk. Strengthening supervision models, refining case selection for trainees, and adopting structured, competency-based learning curves may help mitigate this risk.

Future research should use prospective multicenter designs with standardized reporting of risk factors (axial length, lattice characteristics, PVD status, surgical time, prophylactic laser use, nuclear hardness, required US power during the procedures and supervision models) to enhance comparability across studies and refine evidence-based guidelines for safeguarding patients in training environments.

## Supplementary Information

Below is the link to the electronic supplementary material.

Supplementary Material 1

## Data Availability

The datasets generated and/or analyzed during the current study consisted of patient medical record data and therefore contained confidential clinical information. Deidentified data may be made available from the corresponding author upon reasonable request.
