# Supplementary material for: Incidence and risk factors for retinal detachment after cataract surgery: a comparison between trainee and experienced surgeons
Source: Int J Retina Vitreous. 2026 Jan 8;12:24. doi: 10.1186/s40942-025-00793-z (PMC12870959; doi:10.1186/s40942-025-00793-z)
Supplement: Supplementary file 1 — Supplementary Material 1 [file 40942_2025_793_MOESM1_ESM.docx]

| **Study** | **Country** | **N** | **RRD Incidence** | **Design** | **Key Findings** |
| --- | --- | --- | --- | --- | --- |
| IRIS (2021) | USA | 3,177,195 | 0.21% (1 year) | Retrospective | Population-based risk estimation, no surgeon experience data |
| Daien et al. (2015) | France | 2,680,167 | 0.25% (1 year) | Population-based | Population-based risk estimation, no surgeon experience data |
| Thylefors et al. (2022) | Sweden | 274,108 | 0.29% (5 years) | Population-based | Population-based risk estimation, no surgeon experience data |
| Khatibi et al. (2008) | USA | 3,871 | 0.76% | Comparative | Compared TS vs. ES no statistic significancy |
| Current Study | Brazil | 23,642 | 0.07% (1 year) | Retrospective | Higher RRD with TS (OR 5.78), adjusted for risk factors |

Table 03 – Summarized key data from large-scale population-based studies.
